# Supplementary material for: Home cage-based insights into motor learning and strategy adaptation in a Huntington disease mouse model
Source: PLoS One. 2025 Feb 13;20(2):e0318663. doi: 10.1371/journal.pone.0318663 (PMC11825033; doi:10.1371/journal.pone.0318663)
Supplement: S1 Appendix — (DOCX) [file pone.0318663.s002.docx]

**S2 Appendix. Intra-class correlation and multilevel modeling of daily average hold time.**

To better understand the variability in hold time and its contributing factors, we employed Intraclass Correlation Coefficient (ICC) and multilevel modeling (see methods section) (1,2). ICC measures the proportion of variance attributable to group-level factors (genotype and cage), indicating how much of the variability in performance can be explained by differences between groups, such as genotypes. Multilevel modeling, on the other hand, accounts for both fixed effects (consistent factors like genotype) and random effects (variable factors like individual differences or cage environment).

A high ICC for genotype (19%) suggested that it contributed considerably to the variability in hold time, and this was validated through multilevel modeling. The analysis indicated that a significant difference in motor learning between WT and zQ175 mice persisted while considering cage as a potential factor. Despite a moderate ICC for cage (16%), it did not have a significant relationship with hold time (Table A).

| **Variable value** | **Estimated slope** | **Standard error** | **t values (df = 24)** | **p-value Pr(>\|t\|)** |
| --- | --- | --- | --- | --- |
| Baseline hold time (Intercept) | 0.249** | 0.078 | 0.004 | 0.0038 |
| Time | 0.002 | 0.002 | 1.326 | 0.1971 |
| Genotype | 0.204** | 0.062 | 3.273 | 0.0032 |
| Time:genotype | 0.005* | 0.002 | 2.264 | 0.0328 |
| **Cage [Reference: 8]** |  |  |  |  |
| 1 | 0.014 | 0.125 | 0.111 | 0.9125 |
| 2 | 0.022 | 0.108 | 0.202 | 0.8413 |
| 3 | -0.155 | 0.119 | -1.305 | 0.2041 |
| 4 | 0.068 | 0.106 | 0.643 | 0.5265 |
| 5 | 0.12 | 0.106 | 1.131 | 0.2691 |
| 6 | -0.097 | 0.119 | -0.819 | 0.4211 |
| 7 | -0.177 | 0.108 | -1.645 | 0.1129 |
| 9 | -0.182 | 0.114 | -1.602 | 0.1221 |

**Table A. Multilevel modeling results for daily average hold time.** Multilevel modeling analysis showing the estimated effects of time, genotype, and cage on the daily average hold time of mice performing the lever-pulling task. The table includes the estimated slope, standard error, t values (with degrees of freedom = 24), and p-values for each variable. Significant effects are noted for genotype (**p < 0.01) and the interaction between time and genotype (*p < 0.05), indicating that genotype significantly influences motor learning and that the effect of days in cage on hold time is modulated by genotype. The intercept represents the baseline level of hold time when all other variables are set to zero in the model. Cage 8 serves as the reference category against which the effects of other cages are compared. No significant effects were found for the individual cages.

**References**

1. Snijders TAB, Bosker RJ. Multilevel Analysis: An Introduction to Basic and Advanced Multilevel Modeling. SAGE; 2011. 369 p.

2. Goldstein H, Browne W, Rasbash J. Multilevel modelling of medical data. Stat Med. 2002 Nov 15;21(21):3291–315.
