# Supplementary figures and images for: Home cage-based insights into motor learning and strategy adaptation in a Huntington disease mouse model
Source: PLoS One. 2025 Feb 13;20(2):e0318663. doi: 10.1371/journal.pone.0318663 (PMC11825033; doi:10.1371/journal.pone.0318663)

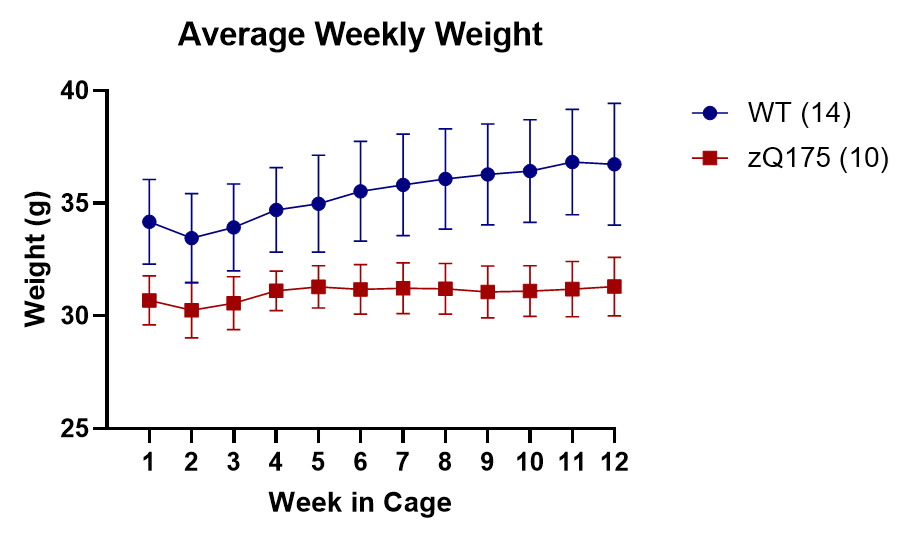

Supplement: S1 Fig — WT mice exhibited a steady increase in body weight over time, while zQ175 mice showed minimal weight gain and maintained a significantly lighter body weight (2–10% lower) compared to WT mice (RM two-way ANOVA, genotype p = 0.116 F(1, 22) = 2.675, weeks p < 0.0001 F(11, 240) = 7.132, interaction p = 0.001 F(11, 240) = 2.797). Data are presented as mean ± SEM. (TIF) [file pone.0318663.s003.tif]
