# Supplementary material for: Home cage-based insights into motor learning and strategy adaptation in a Huntington disease mouse model
Source: PLoS One. 2025 Feb 13;20(2):e0318663. doi: 10.1371/journal.pone.0318663 (PMC11825033; doi:10.1371/journal.pone.0318663)
Supplement: Attachment — Submitted filename: PLoS One_response_to_reviewers_LAR.docx [file pone.0318663.s005.docx]

Dear Editors of PLoS One,

We sincerely thank you and the reviewers for the time and effort spent providing thoughtful feedback on our manuscript. We greatly appreciate the constructive comments and suggestions, which have helped us improve the clarity, robustness, and overall quality of our work. We have made the requested revisions and carefully addressed each point raised by the reviewers.

Our detailed responses to each comment, along with the corresponding edits to the manuscript, are outlined below.

We hope that our revisions meet the expectations of the reviewers and the editorial team, and we look forward to your feedback.

Editor Comments:

Please provide F statistics with numerator and denominator for all ANOVAs.

Thank you for your feedback. We have added the F statistics, including numerator and denominator degrees of freedom, for all ANOVAs in the relevant figure captions where p-values are reported.

For all graphs, please define error bars.

Thank you for your comment. We have defined the error bars in all figure captions as representing the mean ± SEM.

Figure 3:

Please discuss this in a little more detail - Fig 3C shows a quite large reduction in variability in the zQ175 group at 26d approx - what could underlie this?

Thank you for pointing out the notable reduction in variability in the zQ175 group around day 26 in Fig. 3C. Upon closer inspection, this reduction appears to be driven by decreased variability in the performance of individual animals rather than an overall biological shift. During the earlier stages of learning, there are larger fluctuations in the number of trials per entrance, likely reflecting a more variable approach in behavior as the mice adapt to the task. By around day 26, these fluctuations begin to stabilize, as indicated by the smaller error bars, suggesting that the mice may have reached a more consistent engagement pattern.

We opted not to speculate further on potential biological mechanisms underlying this reduction, as we currently lack direct evidence to support specific hypotheses. We have, however, added a brief note in that section to acknowledge this trend:

**The observed reduction in variability appears to arise from a decrease in variability between individual animals rather than a sudden behavioral shift. This stabilization likely reflects a gradual settling into more consistent engagement patterns across the group, rather than an abrupt change in task performance dynamics.**

Fig 3E Are these data from all trials? Have the authors examined at e.g., day 56 versus day 21 and day 1?

Thank you for your question. The data in Fig. 3E represents all trials across the entire testing period. Analyzing the bout size distribution and success rate on a daily basis results in highly variable distributions due to the limited number of data points per day. Instead, we have examined multiple time windows—specifically early (week 1), middle (e.g., week 2-3), and late (week 7-8)—and observed consistent trends in bout size distribution and success rates. The genotype-dependent differences became more pronounced during the middle and late stages, which aligns with the divergence in the number of trials per entrance shown in Fig. 3C.

We believe that since these patterns can already be inferred from Fig. 3C, and would not change the conclusion, adding further data in the results section would introduce redundancy.

Figure 3E - X axis, please consider providing a more explanatory title, e.g., (number of consecutive trials within a single entry)

Thank you for the suggestion. We have updated the X-axis label in Fig. 3E to "Bout Size (Number of Trials Per Entrance)" to provide a more explanatory and intuitive description.

Methods

To adhere to ARRIVE guidelines

--please briefly discuss the group sizes used (e.g., sample size calculations and power analyses).

In our study, we used 10 zQ175 mice and 14 WT mice, consistent with sample sizes commonly used in behavioral studies involving operant conditioning and motor learning tasks.

While a formal power analysis was not performed prior to the study, our group size was informed by prior studies that detected significant motor learning differences in HD models using similar automated home-cage paradigms. Moreover, we applied statistical methods such as multilevel modeling, which are robust to varying sample sizes and account for both within- and between-group variability, increasing the reliability of our results.

We added the following to the methods section:

**Our group size was informed by prior studies that detected significant motor learning differences in HD models using similar automated home-cage paradigms.**

--please briefly discuss housing (were mice of different genotypes housed together)

In this study littermates were housed together, regardless of the genotype. The following was added to the methods section to clarify this:

**Mice were housed at a density of 2 or 3 mice per cage, and including either or both genotypes, as genotypes were not separated.**

**Reviewer #1:** Preclinical research into neurodegenerative diseases, including Huntington’s Disease, depends in part upon motor, cognitive, and social assays performed in disease-modeling animals. However, laboratory animals are highly sensitive to environmental changes and individual handlers, making it challenging to execute these experiments and to interpret results. Automated behavior tasks performed in the home cage can mitigate those challenges, allowing animals to behave in a more natural, unstressed manner, without an observer present.

 Here, the authors convincingly demonstrate the feasibility and value of a novel home-cage behavior monitoring apparatus requiring subject mice to press a lever to receive drinking water. As the animals master the skill, the duration for which they must hold the lever increases, necessitating a flexible problem-solving strategy on the part of the animals. The zQ175 knock-in Huntington’s Disease model mice acquired the initial motor skill similarly to their wild type littermates, but struggled to adapt to the progressive hold-challenge, resulting in fewer, shorter hold bouts. The authors also found that wild type mice, but not HD mice, had more successful trials if they followed a successful “good influencer,” suggesting a possible social learning deficit as part of the mouse’s HD symptomology. Finally, the authors found an increase in fEPSPs in the left hemisphere of wild type mice who had completed this hold-reward experiment compared to the same mouse’s right hemisphere, indicating an increase in plasticity contralateral to the gripping paw. No such difference in lateralization was observed in the HD mice.

 This home-cage system has several compelling advantages. In particular, it allows for social housing, which is profoundly important for animal wellbeing as well as for eliciting the most naturalistic behavior results. This is also helped by the trials being run in the home cage, a familiar and comfortable environment. The RFID scanner detects individuals entering the trial box and presents them with a lever-pull challenge customized to their level of ability, allowing co-housed animals to progress at different rates. This reviewer is satisfied that the authors have a good system in place to minimize the effects of thirst itself on animal health. The tone played for a successful trial allowed the researchers to observe subtle and very interesting social behaviors in animals. This is especially interesting, as few tests exist to measure the social transmission of knowledge among mice.

 There is a punctuation error on line 235, and I encourage the authors to triple-check their punctuation around parentheses. This comment aside, this reviewer feels the authors have demonstrated an interesting and valuable method of measuring motor learning and cognition with the ability to detect learning and coordination deficits in mouse models of neurodegeneration. This method should be of particular interest to the field as it allows for the dissection of subtle social behaviors that would be difficult to capture outside of the mouse’s home environment. This reviewer happily recommends this manuscript for publication without the need for major revisions. The author’s are to be congratulated for a well conducted study and excellently prepared manuscript.

We sincerely thank the reviewer for their detailed and thoughtful review, as well as for their kind words of support and appreciation for our work. We are grateful that the reviewer recognizes the value of our home-cage system and its contributions to the study of motor learning, cognitive adaptation, and social interactions in mouse models of neurodegeneration.

We have carefully reviewed the manuscript once more to ensure that all punctuation, particularly around parentheses, is correct. We appreciate the reviewer bringing this to our attention.

**Reviewer #2:** Many thanks for the opportunity to review this manuscript. The study provides interesting data on assessing a specific pattern of upper limb motor function control in mice, using a home cage lever-pulling device. Furthermore, the study shows some comparative data collected by this device in zQ175 mice as a model of HD and WT mice- showing some valuable differences on the capacity to readjust to specific pulling task that the authors claimed may be associated with the onset of HD phenotype as also assess further by changes on electrophysiological patterns on the striatum. As such, the study is interesting, demonstration further the benefit of such home based and more naturalistic approaches to assess motor dysfunction in mouse models.

 Overall I read the manuscript with interest and I agree with the authors comments on the value of continue developing such technical approaches to study more naturalistic behaviour in prey species like the lab mouse. However, I do think the study has some major challenges once we thoroughly analysed the data and the conclusions that the authors draw form the data. Firstly, the main aim of the study is to validate a new/ revised operant task system, PiPaw2.0 to assess deficits on voluntary movement on these zQ175 HD mice- but the authors already published some early work using similar approaches on a PiPaw system-using the same mice (see reference Woodard CL, Sepers MD, Raymond LA. Impaired Refinement of Kinematic Variability in 821 Huntington Disease Mice on an Automated Home Cage Forelimb Motor Task. J Neurosci.822 2021 Oct 13;41(41):8589–602. While the authors indeed, are now presenting further data- there is no direct comparative analysis on the data acquired by using initial PiPaw system and importantly, how the new 2.0 system is improving the outcomes- note also that looking at the results, many of the data is non-significant which could also query the sensitivity of the system to detect suttled changes. Yes, the reviewer still believes that the data provided in the this paper is of interest. Secondly, there is little information on the study plan and the frequency of study in individual animals- as some of this tasks may be associated with fatigue and exhaustion, it would be good to see a clear experimental design pattern supported by physiological data such as changes on body weight between the animals and groups, any changes on body conditions, any signs of distress on the animals-that could also be an important confounding factor on the overall performance ability of an individual animal. Thirdly, while the authors do a good job on defining many behaviour parameters and their analysis- sometimes it feels like there is an overarching assumption between a specific behaviour and the biological relevance of such functional response. The study mostly provides functional data-with very limited biological data and/or any other type of biomarker data that could aligned the functional data with some clear changes on the brain- atm, this is lacking. There is some informative data on electrophys with some LTP potentiation data- but is this enough to fully associated such changes on the striatum with a HD phenotype on these animals? I would recommend the authors to :

-provide further clarity and experimental data on how this system improved and goes further beyond to the earlier piPaw data (group study in 2021) as this is described as main objective

We sincerely thank the reviewer for their detailed feedback and thoughtful comments. We appreciate the recognition of the study’s novelty and the potential value of naturalistic home-cage-based assessments for studying motor dysfunction in HD mouse models. Below, we address the specific concern regarding the comparison of PiPaw2.0 to our previously published system.

The main differences between our current study and the previous work by Woodard et al. (2021) lie in the HD mouse model used and the task design. The previous study employed symptomatic Q175FDN HD-model mice (Southwell AL et al. *Hum Mol Gen* 2016: doi: 10.1093/hmg/ddw212); these mice are symptomatic by 9 months of age and were tested on the original PiPaw task at 10 -11m performing a task requiring lever pulls within a narrow goal range, without needing to hold the lever for a specified period of time (original PiPaw task). In contrast, our study focuses on early-stage HD using zQ175 knock-in mice at six months of age, a critical time point for detecting subtle impairments in motor learning.

In PiPaw2.0, we introduced a wider goal range and a dynamic hold time requirement, where the necessary hold duration adapts to the mouse's performance. This change enhances task complexity and ensures the difficulty is personalized, encouraging continued engagement and learning over months. Consequently, the average daily number of trials increased, reflecting sustained engagement.

Furthermore, in PiPaw2.0, the software has been extensively refined to eliminate errors and prevent system failures. The updated codebase supports more robust, uninterrupted data collection. Additionally, our annotated dataset is openly accessible, fostering transparency and enabling reproducibility in behavioral modeling studies.

We believe that providing excessive detail on the technical differences between PiPaw and PiPaw2.0 within the main text may risk confusing the reader and detracting from the broader focus of the study. Instead, we have added the following concise comparison in the introduction section to clarify the key advancements without blurring the main narrative.

**This study extends previous work on the PiPaw system by introducing significant changes to the task design and testing a different HD mouse model. Unlike the earlier version, which used a narrow-range lever-pulling task, PiPaw2.0 employs an adaptive hold-time task where the required hold duration of the lever dynamically adjusts based on each mouse’s performance, promoting sustained engagement and continuous learning over months. The updated system also features a more robust codebase, eliminating software errors and enabling reliable long-term data collection, with all annotated data publicly accessible for further analysis.**

a - provides some further physiological/phenotype data on the animals studies including BW, body conditions, pain. distress along with other HD biomarkers of HD disease- I agree that some of these may be challenging some support on biological data would be very useful. Also provide further clarity of cumulative effects on animals during testing to understand the dynamics of study and effects on fatigue.
b -further biomarker/ biological qualification on the WT and HD mouse models so that functional changes are better studies with some biological support. The authors may have some paralel data on the HD mice on what would be their disease phenotype at 6-7 months.

We appreciate the reviewer’s thoughtful suggestion to include additional physiological and biological data to support the functional findings in our study. Below, we address these points in detail:

Physiological Monitoring: As described in the methods section, animal body weights were measured three times per week, and all animals were closely monitored for signs of pain or distress throughout the study. No long-term signs of pain or abnormal behavior were observed in any of the mice. To further support this, we now include a supplementary figure (S3 Fig) showing the average weekly body weights for WT and zQ175 mice.

Body Weight Differences: Our data shows that around six months of age, zQ175 mice were consistently 2–10% lighter than WT mice and exhibited minimal weight gain during their time in the home cage, unlike WT mice. This phenotype has been well-documented in the literature for zQ175 mice (Menalled et al. 2012, Koch et al. 2024) and is consistent with the expected disease progression. Importantly, despite this weight difference, we did not observe any significant differences in key kinematic parameters of movement (e.g., pull speed, maximum pull amplitude, and acceleration), indicating that body size differences do not substantially affect motor strength or task performance.

Invasive Biomarker Considerations: While invasive methods (e.g., tissue sampling or blood collection) are commonly used to measure biomarkers of HD progression, these approaches introduce stress and handling, which would undermine the key advantage of using automated home cages: maintaining a low-stress, experimenter-free environment. We believe that minimizing such stress is essential to accurately study voluntary motor learning in a naturalistic setting.

Supportive Data from Previous Studies: In previous studies using the same zQ175 model at 5–7 months of age (Wang et al. 2023), we characterized head-fixed water-reaching behavior and observed similar impairments in motor learning and control. Additionally, other studies at different ages have shown that automated home-cage paradigms are among the most sensitive methods for detecting subtle phenotypes in HD models (Koch et al. 2024). These findings provide additional support for our interpretation of the results and underscore the robustness of automated systems in capturing early motor deficits.

We added the following paragraph to the revised manuscript, referencing the new supplementary figure:

**It is noteworthy that in line with previous reports on zQ175 mice, we observed a consistent difference in body weight between WT and zQ175 mice starting at approximately six months of age. While WT mice steadily gained weight over the course of the study, zQ175 mice exhibited minimal weight gain and remained 2–10% lighter on average (S3 Fig).**

The results showed many parameters, and many of them of valuable relevance but sometimes there is a far too direct pressumption on who such changes may be linked to the onset /progress of the HD phenotype on the studies mice (QZ174) -so further biological data should be provided to confirm such hypothetized causative effects. This is important to provide further scientific robustness to the fucnctional data- as otherwise the study may be better aligned to a development technical approach with restricted predictability value on the HD models.

We thank the reviewer for their thoughtful critique and for highlighting the importance of avoiding overinterpretation of the functional data. We address these concerns as follows:

Biological Interpretation and Functional Data: We acknowledge that while our study provides functional insights into motor control and adaptation, establishing direct causal links between specific motor impairments and HD progression remains challenging without additional biological data. However, our primary aim was to demonstrate the utility of PiPaw2.0 as a sensitive tool for detecting early motor deficits and adaptive learning challenges in an animal model of HD, rather than solely elucidating underlying mechanisms. Our findings are consistent with previous studies showing that early-stage zQ175 mice exhibit subtle but quantifiable motor deficits, supporting the relevance of our observations (Menalled et al. 2012).

Justification for Hypotheses: Our hypotheses about impaired strategic modulation, movement variability, and social influences are informed by well-established HD literature and previous studies using either similar automated paradigms, or fine motor control tests. The observed inability of zQ175 mice to refine motor performance over time aligns with known corticostriatal dysfunction in HD and parallels behavioral phenotypes observed in human HD patients during early disease stages. Nonetheless, we were extremely cautious in discussing any causation and making definitive claims about the direct causal links, and have revised the conclusion to emphasize that while our findings are suggestive of early HD-related phenotypes, further studies involving additional biological measures are necessary to confirm causative mechanisms.

Predictive Value of PiPaw2.0: The PiPaw2.0 system is not only a technical advancement but also addresses a critical gap in detecting early, subtle motor learning deficits, which are often missed by conventional assays. The prolonged engagement and adaptive task design provide a richer dataset, capturing motor learning over weeks or months. We believe this strengthens the predictive value of our approach, particularly for longitudinal assessments of disease progression in HD models.

We have included the following in the conclusion section of the manuscript:

**While these observations provide valuable insights into early HD-related phenotypes, further studies incorporating additional biological measures will be essential to confirm causative mechanisms and fully elucidate the link between functional deficits and underlying neuropathology.**

There are some other minor issues such as:
 a) any differences between sexes?

While our study was not statistically powered to perform a formal comparison between sexes, we observed the same trend for genotype differences in both males and females. Given this consistency, we combined data from both sexes in our analysis to increase statistical power for detecting genotype-related effects. All details regarding the sex of individual animals, alongside other metadata, are thoroughly annotated in the publicly available dataset to support transparency and further analyses by other researchers.

b? why 6-7months were selected on the study and what would be the differences in body weight curves between the HA and WT line?

We selected the 6–7 month age range because it represents the earliest stage at which subtle motor deficits in zQ175 mice can be reliably quantified (Menalled et al. 2012). This time point is widely regarded as a key early stage for assessing motor impairments and holds promise for evaluating potential drug and treatment interventions before overt symptoms develop.

Regarding body weight differences, starting around 6 months of age, zQ175 mice stop gaining weight and their body size stabilizes at approximately 30 grams, whereas WT mice continue to gain weight over time (S3 Fig). This pattern, as discussed in a previous comment and shown in S3 Fig, has been consistently reported in the literature. The stable body weight of zQ175 mice at this early stage aligns with their disease progression and supports the utility of this age window for detecting early phenotypic differences.

We added the following sentence to the result section to clarify this:

**This age represents an early stage where subtle motor deficits in zQ175 mice can be detected.**

c) what are the WT mice-was is the background for the qZ175 mice?

We thank the reviewer for this question. As mentioned in the methods section, C57BL/6J wild-type (WT) littermates served as controls. To clarify further, we have revised the text to explicitly state that:

**C57BL/6J wild-type (WT) littermates, lacking the expanded allele, served as controls.**

d) please provide info on bedding and cage sizes vs group density per cage as this may affect social interaction and the effects of inter-mouse effects

We appreciate the reviewer’s attention to the potential impact of cage size, bedding, and group density. The cages used in this study measured 7.5” W x 11.5” L x 5” H and were filled with 1/8” pelleted cellulose bedding. Each cage housed 2 or 3 mice. Our multilevel modeling (provided in the supplementary analysis) did not reveal any significant effect of cage density on motor learning outcomes. However, we acknowledge the limited sample size (9 cages total) and cannot rule out subtle effects due to the small number of cages. All relevant details, including group density per cage, are also annotated in the public data repository. To clarify the methods, we added the mentioned details in the methods section.

e)water supply? how was this regulated for the operant conditioning test?

We thank the reviewer for their question regarding the water supply. As described in the methods section, water was delivered via a gravity-fed system connected to a bottle, regulated by a solenoid valve to precisely control the amount of water dispensed during the task. This setup ensured consistent and accurate delivery of water rewards throughout the experiment.

If the reviewer was referring to overall water availability, it is important to note that the task served as the primary source of water. Mice could obtain sufficient water by engaging with the task, and their body weights were monitored regularly to ensure adequate hydration and health (discussed in the methods section).

f) did animals ever reac a 20% body weight loss- this would be considered a severe level of distress so how would you envisage such animals to perform in a non-clinical illness stage and show naturalistic functional outcomes- any animals with a ill-associated homeostasis is very likely that will impact on functional assessments?-how much of the effects detected are seen by such critical physiological status vs the onset/progress of HD phenotype?

We appreciate the reviewer’s concern regarding potential distress due to weight loss and its impact on functional assessments. As described in the methods section, any mouse experiencing a weight reduction > 20% of its pre-home-cage baseline was immediately removed from the study and returned to conventional housing with unrestricted access to water and food to facilitate recovery. Importantly, this only occurred in five instances across the entire study, and all instances were attributed to temporary software failures rather than any underlying physiological issue related to disease or experimental design.

In these cases, after 1–2 days of unrestricted access to food and water, all affected mice returned to their previous activity patterns in the automated home cage, with no visible signs of distress or dehydration. Additionally, their task performance metrics (e.g., number of trials, hold times, kinematic features) returned to baseline levels after reintroduction, indicating no lasting impact on their motor learning trajectories.

We agree that such events could introduce a potential confounder; however, the sparsity of these events relative to the months-long training period and the rapid recovery of affected mice suggest that any influence on our overall findings is minimal. Nonetheless, we acknowledge this limitation and hence have added a note in the methods section to transparently address the potential for transient confounds and emphasize the robustness of the longitudinal data:

**In all five instances, the mice fully recovered within 1–2 days and resumed their previous activity patterns and task performance upon reintroduction to the automated home cage, with no lasting signs of distress or dehydration, though such events could introduce minor confounds in the data.**

g) please provide a better outline methodology on cumulative effects on the animals studied so there is better information associated to risk for fatigue/ exhaustion/boredomness/ motivation.

We thank the reviewer for this comment and agree that providing additional details on how cumulative effects were monitored and mitigated can improve clarity. We interpret this comment as a request for more information regarding how we accounted for potential fatigue, exhaustion, boredom, or changes in motivation throughout the prolonged testing period.

The PiPaw2.0 task was designed to maintain engagement by dynamically adjusting task difficulty based on the individual performance of each mouse. This adaptive design likely mitigates the risk of fatigue or boredom by continuously providing a tailored level of challenge. Importantly, we did not observe signs of disengagement over the study period, as evidenced by the consistent number of daily trials performed by both WT and zQ175 mice. Instead, we believe the task appeared to act as a form of cognitive enrichment, as mice remained motivated to interact with the task for months.

Additionally, prior work using the original PiPaw system showed no signs of disengagement or task aversion in 12-month-old zQ175 mice after 3–5 weeks of training (Koch et al. 2024), supporting the idea that this type of operant task remains engaging and does not induce task fatigue, even in older or more symptomatic animals.

To further address this point, we have added details to the methods and results sections regarding the continuous monitoring of trial counts and the adaptive nature of the task to ensure sustained participation.

The following parts were added to the revised manuscript to clarify these points:

In the methods section: **The PiPaw2.0 task dynamically adjusted the required hold time based on each mouse’s performance to maintain an individualized level of challenge and prevent disengagement due to task monotony or excessive difficulty. Daily trial counts were recorded and monitored over the study period to track engagement.**

In the results section: **Over the course of the study, both WT and zQ175 mice maintained consistent engagement with the task, as indicated by the stable number of daily trials performed. No significant reduction in task participation was observed, suggesting that neither genotype experienced task fatigue or motivational decline.**

Many thanks again for the opportunity to revise this work; as abovementioned, it is of valuable interest but needs further data support and certain clarity in some methodological aspects to strengthen the predictive impact of the functional data provided.

We sincerely thank the reviewer for their time, thoughtful comments, and constructive feedback. We greatly appreciate the detailed suggestions, which have helped us clarify key aspects of our methodology and strengthen the overall impact of our findings. We believe that the revisions made in response to the reviewer’s comments have substantially improved the clarity and robustness of our manuscript.
